# Context-dependent changes in tactile perception during movement execution

**DOI:** 10.3389/fpsyg.2013.00913

**Published:** 2013-12-06

**Authors:** Georgiana Juravle, Francis McGlone, Charles Spence

**Affiliations:** ^1^Crossmodal Research Laboratory, Department of Experimental Psychology, Oxford UniversityOxford, UK; ^2^Department of Systems Neuroscience, Center for Experimental Medicine, University Medical Center Hamburg-EppendorfHamburg, Germany; ^3^Faculty of Science, School of Natural Sciences and Psychology, Liverpool John Moores UniversityLiverpool, UK

**Keywords:** tactile perception, reaching, exploration, active/passive movement, dual-tasking

## Abstract

Tactile perception is inhibited during movement execution, a phenomenon known as *tactile suppression*. Here, we investigated whether the type of movement determines whether or not this form of sensory suppression occurs. Participants performed simple reaching or exploratory movements. Tactile discrimination thresholds were calculated for vibratory stimuli delivered to participants' wrists while executing the movement, and while at rest (a *tactile discrimination task*, TD). We also measured discrimination performance in a same vs. different task for the explored materials during the execution of the different movements (a *surface discrimination task*, SD). The TD and SD tasks could either be performed singly or together, both under active movement and passive conditions. Consistent with previous results, tactile thresholds measured at rest were significantly lower than those measured during both active movement and passive touch (that is, tactile suppression was observed). Moreover, SD performance was significantly better under conditions of single-tasking, active movements, as well as exploratory movements, as compared to conditions of dual-tasking, passive movements, and reaching movements, respectively. Therefore, the present results demonstrate that when active hand movements are made with the purpose of gaining information about the surface properties of different materials an enhanced perceptual performance is observed. As such, it would appear that tactile suppression occurs for irrelevant tactual features during both reaching and exploratory movements, but not for those task-relevant features that result from action execution during tactile exploration. Taken together, then, these results support a context-dependent modulation of tactile suppression during movement execution.

## Introduction

In order to achieve our goals in everyday life, we constantly move and interact with the environment; That is, we frequently perform goal-directed actions. By using simple detection and discrimination paradigms, researchers have provided evidence to suggest that tactile perception changes over the execution phase of goal-directed movements: Tactile sensitivity declines significantly over the execution phase of a movement (Buckingham et al., 2010; Gallace et al., 2010; Juravle et al., 2010; Juravle and Spence, 2011), while tactile stimuli are detected more rapidly (Juravle et al., 2011). Such findings suggest that two psychologically-grounded mechanisms (one of *attentional* facilitation and the other of *suppression*) may work in parallel over the execution phase of a planned movement. The facilitatory attentional effect may help an organism to detect and promptly respond to the incoming sensory novelty, whereas sensory suppression might help an organism to filter out those inputs that are deemed irrelevant to the current task.

That said, the experimental results that have been published to date can be criticized for not taking into account the relevance of the tactile input to the organism's goals: If goal-directed reaching were shown to impair what is felt during the course of a movement, then exploratory movements could provide an answer to the question of whether or not the sensory information arriving at our mechanoreceptors is treated as being of little relevance as soon as we start to move. Alternatively, however, what is felt might be relevant for the goal-directed action and thus necessary to our overall interaction with the environment. To the best of our knowledge, there are no experimental accounts in the literature that have attempted to contrast the characteristics of tactile perception during the execution of reaching movements with the execution of exploratory movements.

The motivation behind the present study therefore relates to a simple paradox: If, when moving, tactile perception is impaired (Dhyre-Poulsen, 1978; Chapin and Woodward, 1982; Chapman, 1988; Cohen et al., 1994), then how can one account for a lack of tactile suppression over the course of an exploratory movement? For example, consider for a moment an ecologically valid task, such as a blind person reading Braille. This task normally involves specific, most often bi-manual, disjoint movements in order to extract useful information from the display (Hughes and Jansson, 1994). Nevertheless, under those conditions in which the participants are required to detect displacements in refreshable Braille displays, tactile suppression occurs (Ziat et al., 2010). Tactile suppression of displacement refers to an inability to detect tactual changes in a moving stimulus, when such changes appear while the fingers are no longer in contact with the specific stimulus, i.e., the tested Braille displays in the above example (Ziat et al., 2010; see also Keyson and Houtsma, 1995). Exploratory hand and arm movements are used in order to identify 3D objects, as well as to distinguish specific characteristics or features of objects in our proximal environment. In daily life, when tactile information is needed in order to achieve our goals, exploratory movements are typically amongst the first to be used. Note that the perceptual-motor process of actively exploring a 2D/3D object is commonly referred to as *haptic perception* (Gibson, 1962; Klatzky et al., 1985; Hatwell et al., 2003; Grünwald, 2008; Lederman and Klatzky, 2009).

Just imagine that you are about to buy a new cashmere sweater: Provided that all of the visual attributes are indistinguishable for two garments, when deciding on the quality of the clothing, you will often move your hands and *feel* the material between your fingers. The movement of one's fingers across the material's surface provides the necessary information with regard to its perceived quality. Such exploratory haptic/tactile behaviors are associated in market research with a *‘need for touch’* that some customers exhibit when evaluating products that they may be interested in purchasing (Peck and Childers, 2003). Indeed, this general liking for haptic input has typically been documented when people interact with clothing, and with novel, or high-quality, products (see Spence and Gallace, 2011; Gallace and Spence, 2014, for reviews). Indeed, possibly mirroring the visual modality, exploratory movements have been metaphorically compared to *‘windows through which the haptic system can be viewed’* (Lederman and Klatzky, 1987, p. 344).

The experiment reported here was designed to test whether the relevance of the tactile/haptic stimulus to a participant's goals modulates the degree to which sensory suppression is observed. For this, we introduced a movement task that is characteristic of haptic perception (i.e., an exploratory movement), together with the goal-directed reaches that have been used previously by researchers (e.g., Juravle et al., 2010). One of the possible implications of the evidence regarding the tactile suppression that occurs during the execution of goal-directed reaching movements (Juravle et al., 2010, 2011) is that touch may appear to be of little relevance to reaching. On the other hand, on a daily basis, exploratory movements are used with the aim of extracting and analyzing important features of the objects that we interact with. Therefore, by comparing the characteristics of tactile perception during the execution of exploratory and reaching movements, it was hypothesized that one could extract the *functional significance* of what is felt while moving.

During the experiment reported here, the participants were seated at a table with their hands and arms on top of the table surface. The participants performed a speeded goal-directed movement as a primary task, together with an unspeeded perceptual task, as a secondary task. The primary movement task involved either simple reaching or exploratory hand movements (i.e., the same as the reaching movements, with the only difference being that contact with the table surface was maintained). For the perceptual task, tactile discrimination thresholds were assessed for vibratory stimuli delivered to the participant's wrists while executing the movement and while at rest. Moreover, in another perceptual task, surface discrimination performance was measured (in a same vs. different task) for the materials covering the surface of the table, during the execution phase of the different movements (i.e., exploration and reaching). This performance measure was intended to evaluate the specificity of exploratory movements. For both perceptual tasks, tactile perception was specifically tested during the movement execution period, where sensory suppression effects have been reported previously (see Juravle et al., 2010, 2011). The vibratory discrimination and surface discrimination tasks could be performed either singly or together, both under active movement and under passive conditions (i.e., when no movement was required, but with the tactile stimulation delivered to the participant's skin by the experimenter, mimicking the surface contact specific to the reaching and exploratory movements).

For the tactile vibratory discrimination task (TD task), the first hypothesis predicted higher discrimination thresholds when participants attempted to report what they felt during the active execution of the movement (both reaching and exploration), as compared to the control rest condition. We hypothesized that if the acuity of a participant's tactile perception were to deteriorate during the course of movement execution, irrespective of the type of movement that is being executed, then no difference in TD task performance between the two active reaching and exploration movements would be observed. However, if exploration brings about an enhancement in what is felt, then tactile sensitivity should be higher for exploratory movements, rather than being diminished, during reaching movements.

The surface discrimination task (SD task) was conceived of as a task that would result in the best behavioral perceptual performance for exploratory movements. Therefore, a significant improvement in surface discriminatory performance was predicted during the execution phase of the exploratory movements, as compared to simple reaches. Moreover, significantly improved performance was expected for active hand movements (i.e., where the participants actively explored the table surface with the aim of gaining some information about its features), as opposed to the passive execution mode, that entailed no voluntary movement. Lastly, for both perceptual tasks, a significant deterioration in participants' performance was expected under conditions of dual-task, as opposed to single task, performance.

## Methods

### Participants

Eight participants (1 male, all right-handed by self-report) took part in this experiment. The mean age was 26 years (ranging from 21 to 29 years). All of the participants reported normal touch and normal hearing. The experimental session lasted for approximately 150 min and the participants received a £20 gift voucher in return for taking part. The experiment was conducted in accordance with the ethical guidelines laid down by the University of Oxford.

### Apparatus

**Figure 1:**
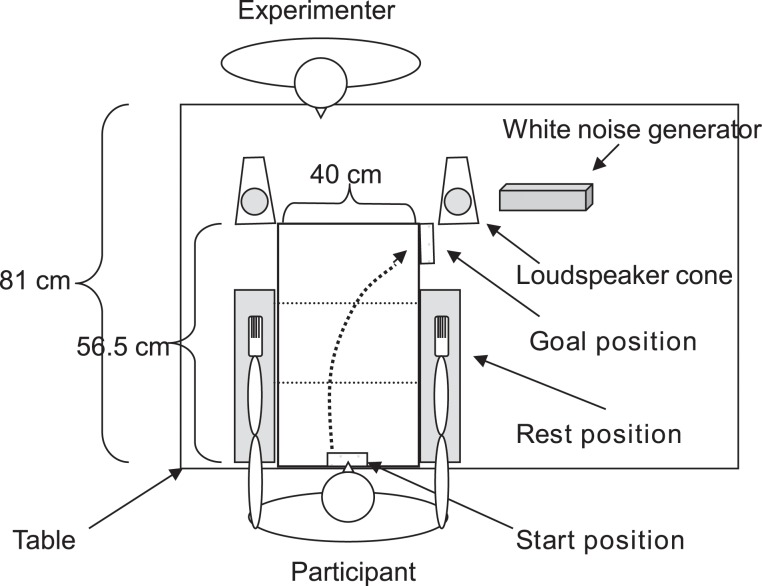
**Experimental set-up**. The participant is depicted with both hands/arms at the resting positions.

The participants were seated at a table (81 cm wide); the experimenter was seated/standing at the other end of the table. A rectangular piece of sponge-like material (24 cm long, 11 cm wide, and 2.5 cm high) was attached to the left side of the table in order for the participants to rest their left hand during the experiment (*left hand resting position*). On the participant's right side, a rectangular piece of wood (21.2 cm long, 17.2 cm wide, and 2.5 cm high) was attached to the table, together with an additional piece of spongy material (same physical measures as for the piece of wood) on top of it (*right hand resting position*). A rectangular object (6.5 cm long, 1.5 cm wide, and 3 cm high; *start position*) was positioned between the two resting positions for the left and right hand at the edge of the table nearest to the participant. The *goal position* was signaled with an identical object placed 19 cm in front of the right resting position. See Figure 1 for a depiction of the experimental set-up.

**Figure 2:**
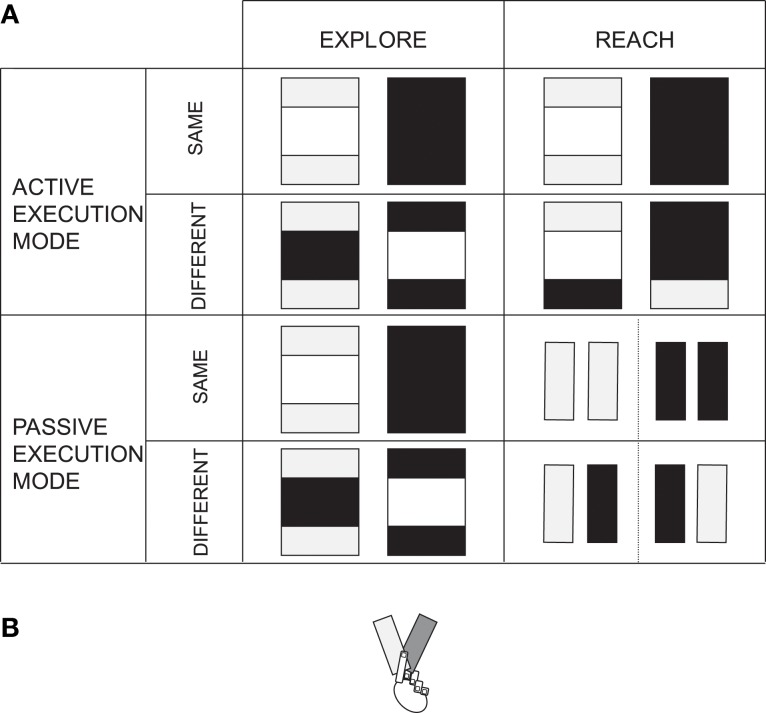
**(A)** Schematic drawings of the types of boards used, split according to the different experimental conditions. Dark shades of gray represent one type of material (i.e., cling film), and lighter shades of gray represent the other material (i.e., tinfoil). **(B)** Schematic depiction of the V-shape arrangement of the boards in experimenter's hand, used for the passive discrimination task.

On each trial, a board (hand made from painting board; 56.5 cm long, 40 cm wide, 0.6 cm high) covered in cling-film, tinfoil, or a combination of the two materials, was placed in front of the participant, between the two resting positions (see Figure 2 for the different types of board that were used in the experiment). Tactors (VW32 skin stimulators, 1.6 × 2.4 cm vibrating surface, Audiological Engineering Corp., Somerville, MA, USA) were attached with tape to the ventral part of both of the participant's wrists and their wrists were then covered in several layers of thin sponge. The participants were blindfolded and wore closed ear headphones (Beyer Dynamic DT 531) for the duration of each block of trials in the experiment. Two loudspeaker cones, one placed on either side of the table, delivered white noise throughout the experimental blocks. Depending on the task, the participants responded by means of two footpedals connected to the computer, as well as vocally, the latter response was entered into the computer by the experimenter.

### Procedure

The experiment involved a speeded motor task (goal-directed reaching movement vs. exploratory movement) and two unspeeded perceptual tasks tactile vibratory discrimination, TD vs. surface discrimination, SD. The motor tasks were designed either as active movements of *only* the right hand, or as passive movements; the left hand was thus always at rest during the experimental trials.

#### Speeded motor task

Prior to the start of each trial, the participants rested their arms at the resting positions. The experimenter ensured that the participants' hands were at the start position and instructed them by saying ‘Ready’ and pressing a key on the keyboard to initiate the trial. At the experimenter's signal, participants brought their hand to the start position (i.e., since they were blindfolded, they learned to feel the start position object positioned at the edge of the table with the side of their index finger). At the start position, the participants were instructed to place their hand over the surface of the board such that they would feel the board's surface with just their index, middle, ring, and little fingers. The thumb, as well as the palmar region of the hand, was not used during this experiment. 500–750 ms after the experimenter's vocal instruction, a short auditory signal was delivered over the headphones (50 ms, 800 Hz). This acted as the Go signal for participants to initiate their movement.

**Figure 3:**
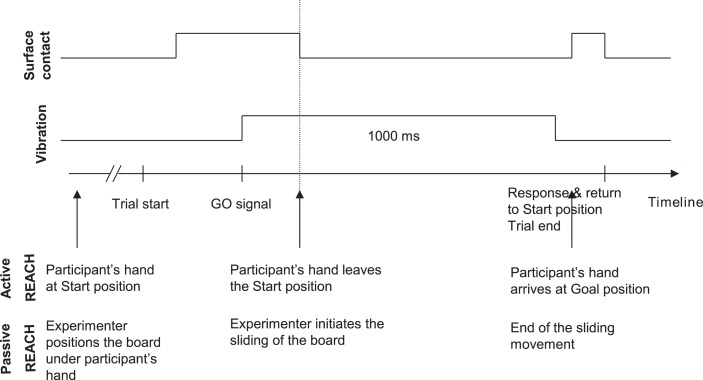
**Schematic depiction of the trial timeline for the REACH movement conditions**. The vibration duration and the contact with the surface of the board are presented for the two types of movement execution modes (active and passive). The EXPLORE movement conditions were identical to the depicted REACH conditions, with the sole exception that in the EXPLORE conditions the contact with the surface of the board was continuous throughout the movement execution phase.

In the *speeded reaching movement condition*, the participants executed an outward movement with their right hand lying across the board's surface. As such, if, at the start of the movement, the participant's forearm was parallel to their torso, it formed an angle of approximately 90° with respect to their torso at the end of the movement. The reaching movement involved a ‘jump’ over the surface of the board, from the start position to the goal position. At the end of the movement, participants touched the object at the goal position with the side of their little finger. Once the goal position was reached, the participants brought their hand back to the right hand resting position (see Figure 3 for a depiction of the trial timeline in the active and passive execution modes of the REACH movement conditions). There was also a control *rest* condition in which no movement of the participant's right hand was required. For this, the participants kept their right hand in the resting position and only performed the perceptual vibratory tactile discrimination task. In the *speeded exploratory movement* condition, at the go signal, participants executed the same outward movement as for reaching movement, this time by keeping contact with the surface of the board until the goal position was reached.

#### Unspeeded perceptual tasks

Two types of perceptual tasks were used to test tactile perception. In the *tactile vibratory discrimination task (TD)*, shortly after the go signal, both of the tactors that the participants wore on their wrists were activated (250 Hz, 12 dB sensation level, 1000 ms). The participants made an unspeeded intensity discrimination response: That is, they had to compare the right hand pulse to the left hand pulse and decide whether the intensity of former was stronger or weaker than that of the latter once they had completed the movement task (and returned their hand to the starting position). The participants were instructed to press one footpedal whenever a stronger pulse was presented to their right hand and the other footpedal whenever the right hand pulse was weaker. Response assignments for the left and right foot-pedals were counterbalanced across participants; see Juravle et al. (2010) for a detailed description of the TD task methodology. In the *surface discrimination task (SD)*, participants had to indicate whether they perceived a change in the material covering the board (by making a same vs. different response).

#### Single versus dual perceptual tasks

Depending on the experimental condition, the two types of unspeeded perceptual tasks could either be performed separately or together. The participants were informed at the start of each block of trials whether it was a single or a dual task block. For the *single task* blocks, the participants only performed a single perceptual task for the entire duration of a block of trials. For example, they performed an exploratory movement on each trial and at the end they either pressed one pedal or the other in order to respond to the quality of the vibratory stimulation, or alternatively, they gave a vocal response with respect to the quality of the surface of the board. In the *dual task* blocks, the participants always performed the vibratory TD task as a first perceptual task. In randomly chosen trials, after they had made their pedal response for the first perceptual task, the experimenter requested their response to the second SD task. Once again, the participants responded vocally and the experimenter entered their response into the computer. Within an experimental dual task block, the participants did not know in advance which trials would require an additional perceptual response.

#### Active versus passive execution modes

Two types of execution mode were used: active and passive. The active mode corresponds to the (active) speeded reaching or exploration movement executed by the participants, presented in Section *Speeded motor task*. In contrast, the passive execution mode was introduced as a means of mimicking the participant's movement, without the actual movement of the limb. In this respect, participants always kept their hands at the resting positions. At the start of each trial in the *passive exploration* condition, the experimenter placed the board underneath the participant's fingertips. At the Go signal, the experimenter touched their fingertips with the board and then slid the board at a constant speed along the surface of their fingertips. In the *passive reaching* condition, the experimenter used two smaller boards, one covered in cling film, and the other in tinfoil (see Figure 2A). The experimenter kept the boards with their lower corners superimposed in her left hand, such that they formed a V at an angle of about 45° (see Figure 2B). At the beginning of the trial, depending on the condition, the experimenter positioned one of the ends of the board underneath the participant's fingertips. At the Go signal, the experimenter gently touched the participant's fingertips with the prepared end, after which, the experimenter moved the V, such that the other end could be positioned underneath and touch the fingers as well. This is an example of the procedure for a trial that required the use of different materials. When the same material was to be used for the passive reaching condition, the experimenter simply paused shortly after the first touch and then performed the second touch with the same material. Once the participants had made their response, the experiment progressed onto the next trial following a random inter-trial interval of 1500–2500 ms. The experimenter read the trial definition on the computer screen at the very beginning of each trial and changed the table board according to the up-coming condition.

### Design

The experiment consisted of nine blocks of trials. Each block corresponded to an experimental condition. The manipulated variables were: type of movement (reaching vs. exploration), execution mode (active vs. passive), and type of perceptual task (single vs. dual). Therefore, it was a 2 × 2 × 2 design; the ninth block consisted of the control rest condition for the TD. The order in which the various experimental blocks were presented was counterbalanced across participants (see Table 1 for a summary of the experimental design). Note that given the psychophysical task utilized, it was appropriate to use a small sample size to extensively test the various experimental dimensions (Quinn and Watt, 2012).

**Table 1:** **Summary of experimental design**.

**Passive execution mode**					**Active execution mode**			
**Single task**			**Dual task**		**Single task**		**Dual task**	
TD rest	SD explore	SD reach	SD + TD explore	SD + TD reach	SD explore	SD reach	SD + TD explore	SD + TD reach

SD, surface discrimination task and TD, tactile discrimination task.

The design of the TD task was detailed previously in Juravle et al. (2010, 2011). The SD consisted of 28 trials per block. For half of the trials, boards consisting of the same material plates were used (i.e., 7 trials with cling film-only boards, and 7 trials with tinfoil-only boards). For the other half of the trials, the boards were made of different materials (i.e., 7 trials in which the covering material of the board changed from cling film to tinfoil, and 7 trials in which the material changed from tinfoil to cling film). The SD-only blocks consisted of the 28 randomly intermixed trials. The dual-task blocks had the 28 SD trials randomly intermixed amongst the TD trials. Given the psychophysical procedure used to determine the perceptual threshold in the TD task, the total number of trials needed for the completion of the TD conditions varied between participants; the maximum number of trials per staircase/condition was set to 120.

### Data analysis

Perceptual thresholds were calculated for the TD data, together with percentages of correct responses for the SD data. Depending on the experimental question, several analyses were performed that involved the use of repeated measures analyses of variance (ANOVAs).

#### TD data analysis

For the TD analysis, three different ANOVAs were performed. In order to investigate whether the execution of the movement interfered with what participants felt, a first ANOVA was conducted with the factor condition (rest vs. active exploration vs. active reaching). The next step involved investigating whether it was not only the movement that affected tactile sensitivity, but also the concomitant tactile input delivered to a resting hand. For this, a second ANOVA was performed with the factor of task type (TD at rest vs. TD at rest plus passive exploration vs. TD at rest plus passive reaching). The third analysis was designed to investigate whether the movement type and the mode of movement execution gave rise to differential changes in tactile perception. For this, a 2 × 2 repeated measures ANOVA was conducted with the factors of movement type (exploration vs. reaching) and execution mode (passive vs. active).

Lastly, for the dual-task conditions, bivariate correlations were conducted between the data from the two perceptual tasks, TD task and SD task, performed together under the dual task conditions. In order to investigate whether a relationship between the distribution of tactile thresholds in the TD task and performance in the SD task arose from the dual task situation, these primary correlations were followed by further partial correlations between the data from the two perceptual tasks performed together, while controlling for the data from the single task SD condition.

#### SD data analysis

For the SD analysis, one general 2 × 2 × 2 repeated measures ANOVA was performed with the factors of movement type (exploration vs. reaching), execution mode (passive vs. active), and task type (single vs. dual). Furthermore, bivariate correlations were conducted for each type of movement, between the data collected under single and dual task conditions. In order to investigate whether a relationship between the distribution of correct responses in the SD task and that of the tactile thresholds in the TD task could be explained by the simultaneous performance of the TD task, separate partial correlations were conducted between the same data while controlling for the variable TD at rest.

## Results

### TD task results

**Figure 4:**
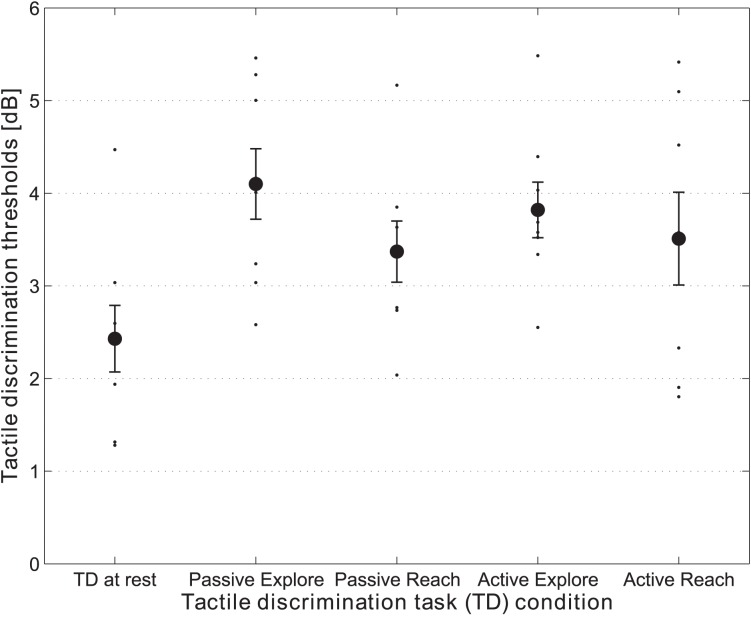
**Scatter-plots of the individual threshold data together with the means for the TD task conditions**. Vertical error bars represent the standard errors of the means.

**Figure 5:**
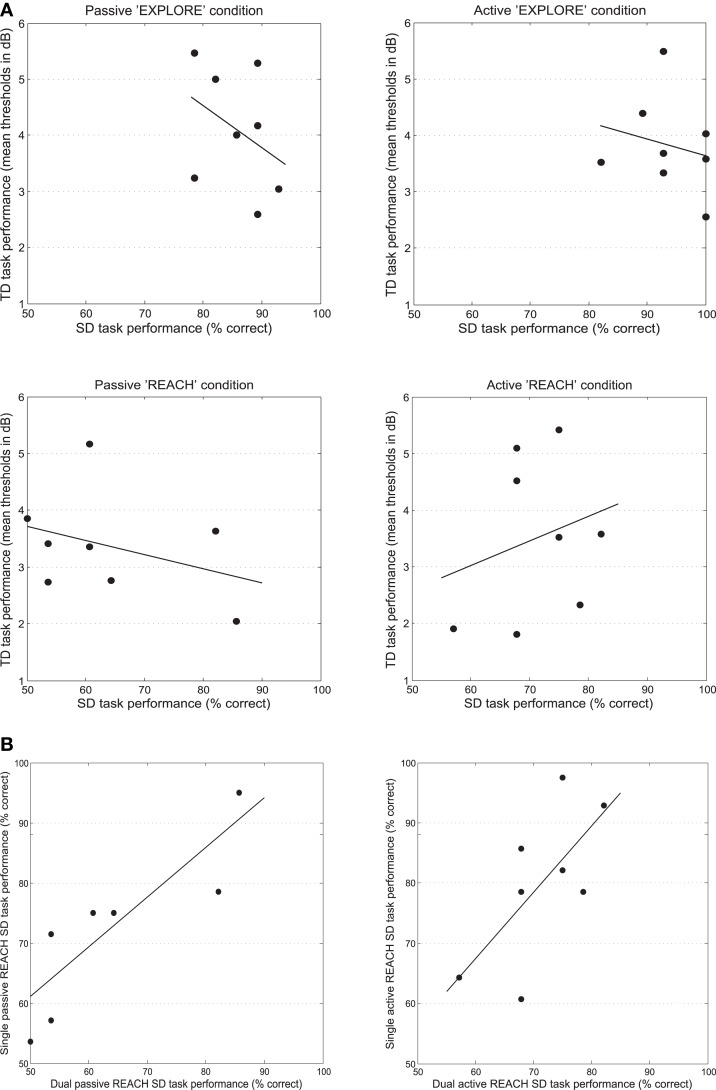
**Scatter plots of TD task performance (in dB) plotted against the performance in the SD task (% correct) for all of the dual task conditions**. The dots represent the mean threshold data from individual participants **(A)**. Scatter plots of the single task plotted against the dual SD task performance for both the active and passive REACH conditions. The dots represent the mean % correct SD task data from individual participants **(B)**.

Mean thresholds and individual data from all participants are presented in Figure 4. In Figure 5A, the TD threshold data are plotted against the performance in the SD task for all of the dual-task conditions in the experiment.

As expected for the TD task, the results indicated that tactile perception was impaired during movement, *F*_(2, 14)_ = 8.26, *p* = 0.004. The participants were significantly less sensitive in discriminating the quality of tactile stimulation while they were performing the active exploration movement (*p* < 0.001), as well as the active reaching movement (*p* = 0.045), as compared to the control rest condition, where no movement was performed. No significant difference was observed between the mean thresholds of the two active movement conditions, exploration and reaching (*p* = 0.451).

With regard to the TD task when performed at rest, the results indicated a significant effect of the type of task, *F*_(2, 14)_ = 18.95, *p* < 0.001. That is, participants were significantly more sensitive to the quality of tactile stimulation for the TD at rest, as compared to those conditions in which the same task was performed while receiving ‘passive exploratory input’ (*p* = 0.003), or ‘passive reaching input’ (*p* = 0.001). Moreover, a significant difference was observed between the mean thresholds in the two passive dual task conditions: That is, tactile thresholds were significantly elevated (i.e., performance was significantly poorer) for the passive exploratory input, as compared to the passive reaching input (*p* = 0.030).

Lastly, there was no significant main effect of the mode of movement execution, *F*_(1, 7)_ <1, n.s., movement type, *F*_(1, 7)_ = 4.59, *p* = 0.069, nor any interaction between the two variables, *F*_(1, 7)_ <1, n.s., when comparing the data from the two movement types, across the two movement execution modes. No significant correlations were found between the distribution of the TD and that of SD performance under dual task conditions.

### SD task results

**Figure 6:**
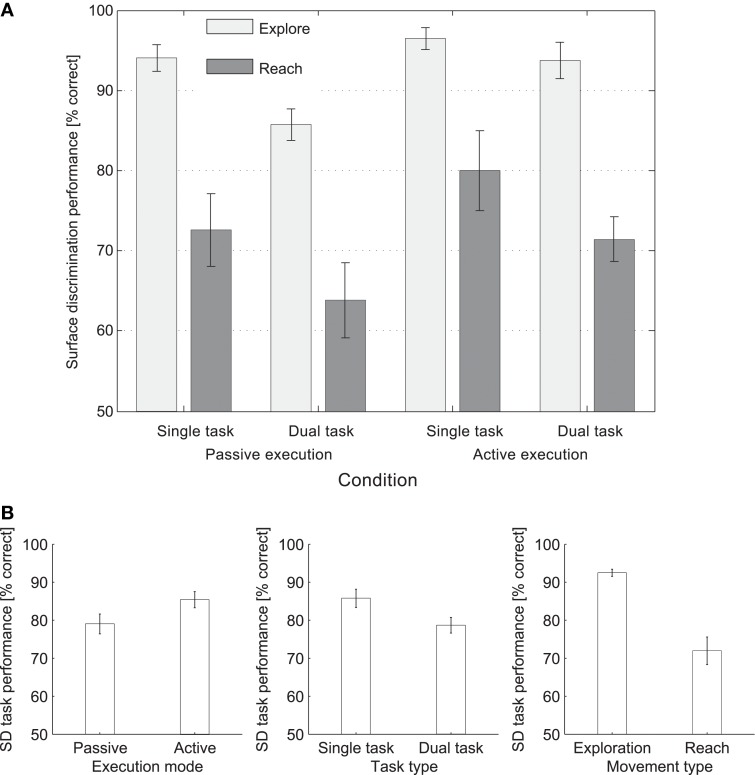
**(A)** Percentages of correct responses for the different conditions in the SD task. **(B)** Significant main effects of Execution mode, Task type, and Movement type in the SD task.

Percentages of correct responses for all the experimental conditions are presented in Figure 6A. The results highlighted significant main effects of all of the experimental variables: movement type, *F*_(1, 7)_ = 44.71, *p* < 0.001, execution mode, *F*_(1, 7)_ = 10.19, *p* = 0.015, and task type, *F*_(1, 7)_ = 36.29, *p* = 0.001. As such, participants' SD discrimination performance was significantly better under conditions of active movement as compared to passive movement, single tasking as compared to dual tasking, and while exploring the surface of the board, as compared to reaching movements; see Figure 6B for a depiction of the significant main effects. No significant interactions between the experimental variables were found.

For the passive execution mode, a positive correlation was demonstrated between the Reach SD task performance under conditions of single and dual tasking, *r* =0.853, *p (one-tailed)* = 0.007, *R*^2^ = 0.727. When controlling for the performance in the TD when performed at rest, the same strong correlation between the two variables was observed, *r* =0.851, *p (one-tailed)* = 0.008, *R*^2^ = 0.724, suggesting that the variance found in the passive SD reaching condition could not be explained by the additional TD task.

Similarly, for the active execution mode, a positive correlation was demonstrated between the Reach SD task performance under conditions of single and dual tasking, *r* = 0.679, *p (one-tailed)* = 0.032, *R*^2^ = 0.461. When controlling for the performance in the TD when performed at rest, the same positive correlation between the two variables was observed, *r* = 0.683, *p (one-tailed)* = 0.045, *R*^2^ = 0.466, suggesting that the variance found in the active SD reaching condition could not be explained by the performance of the additional task. See Figure 5B for plots of the significant correlations. No other significant correlations were found.

## Discussion

The present experiment was designed to investigate, at a behavioral level, *whether* and *how* tactile suppression manifests itself during specific hand movements. To this end, a demarcation between *movement types*, as well as between *modes of movement execution*, was utilized in order to obtain a comprehensive view regarding what actually happens to tactual information during movement. Here, some of the methodological issues raised are considered, followed by a discussion of the results of each perceptual task, and ending with some general conclusions.

### Methodological considerations

#### Speed of movement

A first factor that it was not possible to control and which really needs to be taken into account is the speed of movement since it is known that the degree of experienced tactile sensory suppression decreases as the speed of the movement decreases (Angel and Malenka, 1982; Schmidt et al., 1990). Following on from this, it has been argued that when performing exploratory movements, participants may adjust the speed of their hand movements such that the desired features of the surface are more easily assessed (Chapman, 2009). As such, a slowing of the hand movement occurs during exploration, as opposed to the more rapid hand movement that occurs during reaching actions. On these grounds, it has been suggested that the known attenuation of tactile perception will occur for faster movement speeds, but not for the slower ones that are typically used in exploration.

Indeed, in this respect, a recent study tested the critical speed of movement needed for sensory suppression to occur (Cybulska-Klosowicz et al., 2011). Participants performed a simple inward movement of their right hand, with the speed of the movement entrained to a signal presented on an oscilloscope; brief electrical stimuli were delivered to participants' middle fingers during the movement execution period, or in a control condition performed at rest. Participants had to make unspeeded perceptual judgments regarding the presence or absence of the weak tactile stimulation. In a blocked design, speeds ranging from very slow through to ballistic were tested. For each participant, the critical speed at which tactile detection dropped to chance level was calculated. Not surprisingly, for all of the participants, the critical speed exceeded 200 mm/s, with a mean of 472 mm/s. Such a result was taken to show that tactile suppression occurs with movement speeds outside the typical range of 50–200 mm/s that are used in exploration (Essick and Whitsel, 1985).

Moreover, the participants in Cybulska-Klosowicz et al. study (2011) were asked at the end of each block of trials whether they would use the respective tested speed for an exploratory movement. Most of the participants indicated that they would use speeds slower than 200 mm/s for exploration, but at the same time, a significant proportion indicated faster speeds as being appropriate for an exploratory movement. The authors explained these results as follows: The participants did *not* have surface contact for the movement tested in their experiment which made the evaluation of the speeds difficult. This begs the question of what exactly happens with tactile suppression when tested with specific exploratory movements, a question that was specifically addressed in the experiment reported here.

#### Locus of tactile stimulation

Tactile perception was measured by means of two perceptual tasks: one TD task measuring tactile discrimination thresholds at participants' wrists, and another SD task, measuring surface discrimination at the participants' fingertips. One could criticize the present design on the grounds that we did not measure tactile perception in both tasks at the same skin location. However, considering the perceptual tasks that we were interested in, having different skin locations to measure tactile performance was the most practical solution. In support of this, tactile suppression has nevertheless been shown to “invade” the moving limb, such that when moving a finger, a decrease in what is felt is also present for the surrounding regions of the arm (see Williams et al., 1998).

#### Dual-task effects in both TD and SD perceptual tasks

The two tasks used in the present study to measure tactile discrimination performance could either be performed alone, or else together, within the same experimental block. Given this experimental design, some deterioration in performance was to be expected and was indeed detected when comparing conditions of single versus dual tasking: Participants demonstrated significantly higher tactile thresholds (i.e., poorer discrimination performance) when the TD task was performed together with the SD task, under both active and passive execution modes for the tested movements. Conversely, participants' performance in the SD task was significantly worse when this accompanied the TD task, as compared to those conditions in which the participants only performed a single task.

### Discussion of TD task performance

Given previous experimental results on tactile suppression during the execution of goal-directed reach-to-grasp movements (Juravle et al., 2010, 2011), it was hypothesized that increased tactile thresholds (i.e., poorer performance) would be observed for the *active* goal-directed reaching movements, as well as for exploratory movements, as compared to thresholds measured in a control no-movement condition. This hypothesis was confirmed: Participants' performance deteriorated during movement execution (i.e., tactile suppression was observed). Moreover, the two types of active hand movements (exploratory or simple reaching movements) resulted in a similar deterioration of what was felt during movement, thus indicating that irrespective of the type of movement performed, tactile perception was affected.

Furthermore, tactile perception was significantly impaired during the *passive* execution modes for both movements tested (i.e., participants kept their hand at rest, while the experimenter touched it such that it mimicked the contact with the table surface from the active conditions), as compared to the control rest condition (see also Williams and Chapman, 2000). Such a result could indicate that not only does actively moving the hand trigger tactile suppression, but also that the additional *distracting* tactile input provided to a resting hand leads to the same suppressive effect on tactile perception (Juravle and Spence, 2011). This distracting factor, one that gives rise to impaired performance in those conditions in which the two perceptual tasks were performed together, could, of course, be taken as a dual-task effect: TD task performance was impaired when participants performed the concomitant SD task. In a similar vein, the necessity for participants *to divide* their attention between the two tasks could have contributed to the clear deterioration in TD task performance, when the additional tactile SD was performed. However, in the case of the passive movement execution mode, tactile discrimination thresholds were significantly higher for the exploratory movements, as compared to the reaching movements. Such a result hints at the possibility that *distraction* was the more likely explanatory mechanism for the present results. Note, though, that the passive exploration task involved a sustained contact between participants' fingers and the experimental board, as opposed to the passive reaches that involved two temporally segregated touches, delivered by the experimenter. In this respect, the time given to the participant during the trial to extract the needed tactile discrimination cues in this experiment (e.g., tactile memory, Gallace and Spence, 2009) could be taken as an additional factor accounting for the significant difference between tactile sensitivity measured under conditions of passive exploration and passive reaching.

Lastly, when comparing the data from the two types of movement, across the two modes of movement execution, no significant difference in tactile sensitivity was observed for the two execution modes and the two movement types, nor was any interaction observed between the two variables. The latter result is particularly important since it underlines the fact that tactile sensitivity is similarly affected by the two types of movement, exploration and reaching. Such a finding could be taken to account for the fact that (i) either participants did not adjust the speed of their movement, in order for the exploratory movement to be performed appropriately (see Cybulska-Klosowicz et al., 2011); or else (ii) if participants adjusted their speed (i.e., they slowed down their movement), then speed alone does not delineate between a suppressed state of tactile perception and a non-suppressed state. Following on from this, a discussion of the *movement type relevance* for what is felt is needed. This possibility is considered in the next section.

### Discussion of SD task performance

Since the goal of tactile exploration is to gain information concerning the characteristics of objects that we come into contact with (i.e., the surface of the experimental board in this case), the first prediction with respect to the SD task entailed significantly higher discrimination performance for exploratory movements, as opposed to the simple reaching movements. This hypothesis was confirmed: Participants were significantly better at discriminating between the two materials covering the table surface when they performed exploratory movements, as compared to simple reaches. This is an important result, since it highlights *the relevance* of the movement chosen when measuring tactile performance during movement (Knecht et al., 1993). This result is further strengthened by the positive correlations found for both active and passive reaching movements between the SD task performance under conditions of single and dual tasking: If performance declined for the single reach SD task, it also declined for the dual reach SD task (performed together with the TD task), and the variance in either of their distributions was not explained by the additional perceptual task. Therefore, with respect to the question of the relevance of the task, it appears that the goal-directed reaches may not be the ideal movements with which to investigate tactile perception enhancements during movement execution.

Furthermore, as expected, participants' performance was significantly higher when actively performing the tested movements, as opposed to the passive execution condition. Note that for simple tactile features of objects (i.e., tactile roughness discrimination thresholds), the movement execution mode (active or passive) was not found to make a difference with respect to the performance on the task (Hsiao et al., 1993; Jones, 2009). These studies, however, have only used the natural exploratory movements in their design. In the present study, where exploration was contrasted with reaching movements, when performing a purposeful movement (i.e., moving the hand on the surface of the board in order to get tactual information about it), performance was significantly better as compared to simply receiving the same tactual information, in the absence of overt movement. Such a result thus highlights the importance of purposeful movement for tactile perception.

In conclusion, it would appear that for unspeeded perceptual tasks involving the delivery of tactile stimuli during the execution of simple reaching movements, as well as exploratory movements, a dichotomy based on *sensory-relevance* for movement is apparent: The characteristics of tactile stimulation that are not relevant to the motor task at hand will most likely be suppressed, in order to highlight other incoming valuable sensory information. However, tactile information that is relevant to the motor task at hand, such as that used in active exploration, will be enhanced. From this perspective, the attentional/suppressive influences on what is felt during movement could thus be regarded as being *context-dependent* (Chapin and Woodward, 1982; Fanselow and Nicolelis, 1999; Ferezou et al., 2007).

### Conflict of interest statement

The authors declare that the research was conducted in the absence of any commercial or financial relationships that could be construed as a potential conflict of interest.
